# *Escherichia coli* ASKA Clone Library Harboring tRNA-Specific Adenosine Deaminase (*tadA*) Reveals Resistance towards Xanthorrhizol

**DOI:** 10.3390/molecules200916290

**Published:** 2015-09-09

**Authors:** Yogiara, Dooil Kim, Jae-Kwan Hwang, Jae-Gu Pan

**Affiliations:** 1 Department of Biotechnology, Yonsei University, 50-Yonsei-ro Seodaemun-gu, Seoul 120-749, Korea; E-Mail: yogiara@yonsei.ac.kr; 2 Faculty of Biotechnology, Atma Jaya Catholic University of Indonesia, Jalan Jenderal Sudirman 51, Jakarta 12930, Indonesia; 3 Superbacteria Research Center, Korea Research Institute of Bioscience and Biotechnology (KRIBB), 111 Gwahangno, Yuseong, Daejeon 305-806, Korea; E-Mail: dikim2006@daum.net

**Keywords:** food-grade antimicrobial compounds, xanthorrhizol, tRNA-specific adenosine deaminase

## Abstract

Xanthorrhizol is a potent antimicrobial compound isolated from the rhizome of *Curcuma xanthorrhiza*. However, the mechanism of xanthorrhizol action is unknown. To screen for probable target(s), we introduced the ASKA pooled-plasmid library into *Escherichia coli* W3110 *imp4213* and enriched the library for resistant clones with increasing concentrations of xanthorrhizol. After three rounds of enrichment, we found nine genes that increased xanthorrhizol resistance. The resistant clones were able to grow in LB medium containing 256 µg/mL xanthorrhizol, representing a 16-fold increase in the minimum inhibitory concentration. Subsequent DNA sequence analysis revealed that overexpression of *tadA*, *galU*, *fucU*, *ydeA*, *ydaC*, *soxS*, *nrdH*, *yiiD*, and *mltF* genes conferred increased resistance towards xanthorrhizol. Among these nine genes, *tadA* is the only essential gene. *tadA* encodes a tRNA-specific adenosine deaminase. Overexpression of *E. coli* W3110 *imp4213* (pCA24N-*tadA*) conferred resistance to xanthorrhizol up to 128 µg/mL. Moreover, overexpression of two *tadA* mutant enzymes (A143V and F149G) led to a twofold increase in the MIC. These results suggest that the targets of xanthorrhizol may include *tadA*, which has never before been explored as an antibiotic target.

## 1. Introduction

Food material can be a promising resource for antimicrobial discovery. Xanthorrhizol, the main compound in the rhizome of Java turmeric (*Curcuma xanthorrhiza*), is known to exhibit antimicrobial activity against Gram-positive bacteria and against several Gram-negative bacteria, such as *Bacillus cereus*, *Staphylococcus aureus*, *Streptococcus mutans*, *Salmonella typhimurium* KCCM 11862 and *Vibrio parahaemolyticus* [1,2,3]. Furthermore, xanthorrhizol shows rapid bactericidal activity against *S. mutans* [1]. Xanthorrhizol was recently used to target the enoyl-ACP reductase (FabI) of *E. coli* [4]. However, targeting FabI, which is a bacteriostatic target [5,6], does not explain the rapid killing mode of action of this compound, thus implying that screening for other candidates may reveal additional xanthorrhizol targets.

Encompassing a complete set of *E. coli* genes, the ASKA clone (−) library (A complete Set of *E. coli* K-12 ORF Archive) comprises 4122 clones [7]) that are suitable for, but not limited to, systematic functional genomics study, including DNA microarrays and protein expression, protein localization, and protein–protein interaction studies [8]. The ASKA clone library has also been used to confirm or screen for antimicrobial targets [9,10,11]. Couce *et al.* used the ASKA clone library to overexpress genes that may confer resistance to fosfomycin [9]. Genome-wide screening of the ASKA clone library in LB medium containing fosfomycin resulted in the identification of a clone harboring pCA24N-*murA* as the sole survivor [9]. A study to identify triclosan targets using ASKA library enrichment revealed multiple triclosan resistance genes [11].

In this study, we used the ASKA clone (−) library to identify gene(s) that could increase resistance to xanthorrhizol. Unfortunately, xanthorrhizol is less active against several Gram-negative bacteria, including *E. coli* [3]. To overcome this limitation, we used *E. coli* W3110 *imp4213* as a host for the ASKA plasmid library. This *E. coli imp* strain exhibits increased membrane permeability [12,13], rendering it more susceptible to ampicillin, carbenicillin, bacitracin, erythromycin, novobiocin, and rifampin [14]. We also found that *E. coli* W3110 *imp4213* was susceptible to xanthorrhizol at a minimum inhibitory concentration (MIC) of 16 µg/mL. By using this ASKA library, we identified another xanthorrhizol target, *tadA*, a tRNA-specific adenine deaminase.

## 2. Results and Discussion

Wild-type *E. coli* W3110 cannot be killed by xanthorrhizol (MIC > 512 µg/mL). To use *E. coli* as a model for xanthorrhizol resistance, we used the *E. coli* W3110 *imp4213* strain in our laboratory collection as a host for the ASKA pooled-plasmid library. *E. coli* W3110 *imp4213* is susceptible to xanthorrhizol, with an MIC of 16 µg/mL.

### 2.1. ASKA Library Enrichment and Screening for Xanthorrhizol Resistance

**Figure 1:**
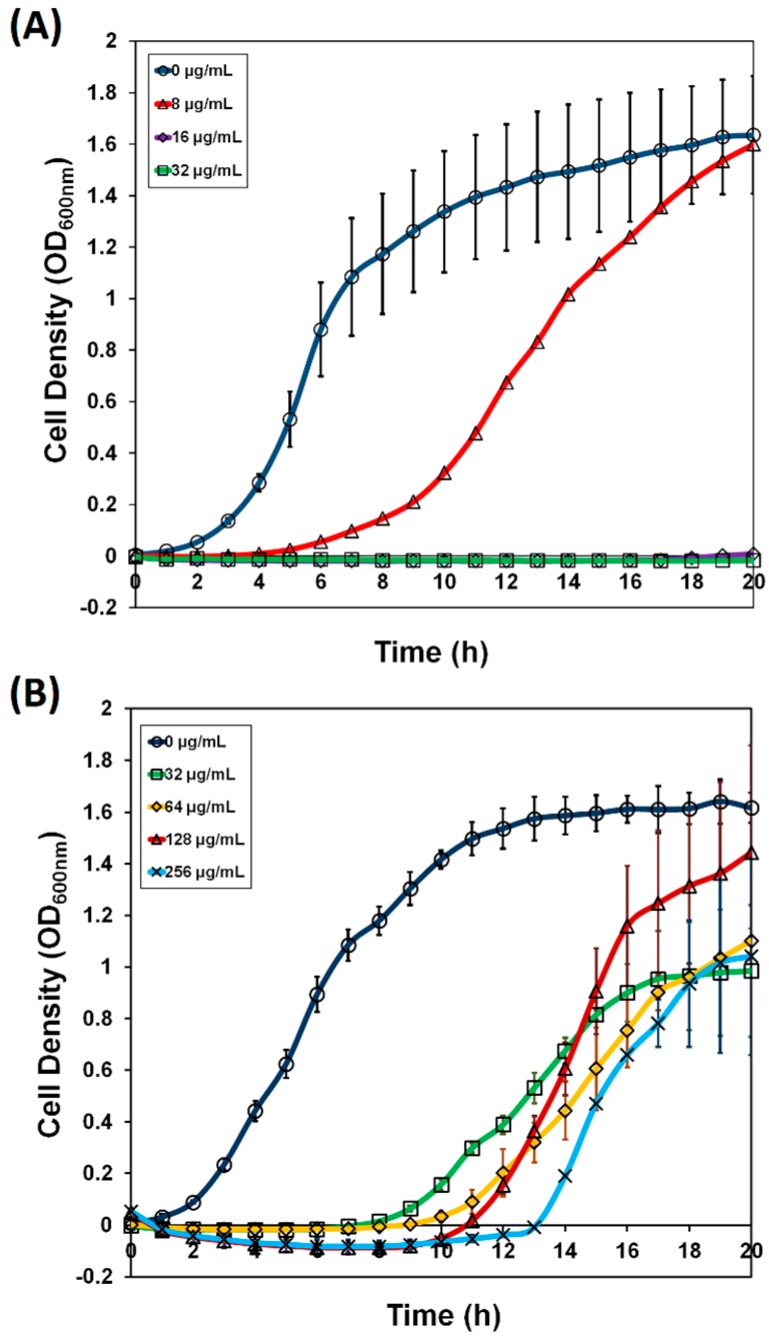
Growth of the *E. coli* W3110 *imp4213* (pCA24N) and *E. coli* W3110 *imp4213* ASKA pooled-plasmid library culture in Luria broth media supplemented with chloramphenicol (30 µg/mL) and with various concentrations of xanthorrhizol. Growth was monitored for 20 h in an automatic incubator (TVS1 Bio-Photorecorder, Advantec, Shiga, Japan). (**A**) *E. coli* W3110 *imp4213* (pCA24N) was grown in the present of xanthorrhizol 0, 8, 16 and 32 µg/mL. The growth was inhibited at 16 µg/mL; (**B**) *E. coli* W3110 *imp4213* ASKA pooled-plasmid library was grown in LB media with various concentrations of xanthorrhizol after three rounds of enrichment. The xanthorrhizol concentrations used were 0, 32, 64, 128, and 256 µg/mL. The library clones that grew after the delay exhibited resistance towards xanthorrhizol.

In this study, we used a complete set of ORF clones from the *E. coli* ASKA library to screen for gene(s) that confer resistance to xanthorrhizol. This is a plasmid-based genomic screening system that was modified from the protocol used for the triclosan target screen [11]. Cells that can grow at high concentrations of xanthorrhizol may bear plasmids containing genes that confer xanthorrhizol resistance. Such clones would likely out-compete others in the culture. The library grown in the absence of xanthorrhizol was used as a growth control. After three rounds of enrichment, clones from *E. coli* W3110 *imp4213* harboring the ASKA pooled-plasmid library were isolated from the cultures grown with the highest concentrations of xanthorrhizol (Figure 1). The library culture showed delayed growth in LB medium supplemented with xanthorrhizol, compared to *E. coli* W3110 *imp4213* harboring an empty plasmid pCA24N (Figure 1). After the final round of enrichment, the clones could grow at a xanthorrhizol concentration of 256 µg/mL within 20 h of inoculation.

A total of 107 plasmids were isolated from the enriched library clones. Sequencing analysis of the isolated plasmids revealed nine different genes that may be related to xanthorrhizol resistance (Table 1). An ASKA clone harboring the *tadA* gene dominated the enriched culture (71 out of 107 isolated plasmids, or 66.4%), followed by the ASKA clones carrying *galU* (11.2%), *fucU* (7.5%), *ydeA* (5.6%), *ydaC* (3.7%), *soxS* (1.9%), *nrdH* (1.9%), *yiiD* (0.9%), and *mltF* (0.9%). Among these nine *E. coli* genes, only *tadA* is classified as an essential gene [15]. *tadA* encodes tRNA-specific adenosine deaminase A.

The *tadA* gene encodes a tRNA-specific adenosine deaminase that specifically converts A_34_ to I_34_ in bacterial tRNA^Arg2^ [16]. The *tadA* gene is known to be essential in *E. coli* MG1665 [16,17,18] and *Shewanella oneidensis* MR-1 [19]. However, this gene is noted as non-essential in *Bacillus subtilis* 168 and *Pseudomonas aeruginosa* UCBPP-PA14 in the Online Gene Essentiality Database [20]. The *tadA* protein is conserved (>83%) among Gram-negative and Gram-positive bacteria, and yeast [16]. Prokaryotic *tadA* shares amino acid sequence similarity with the Tad2P enzymes in humans, *Drosophila*, and *Saccharomyces* [21]. Tad2P deaminates adenosine, but its deaminase domain is similar to that of the cytidine deaminase (CDA) superfamily [22]. However, *tadA* is specific for prokaryotic tRNA substrates and it specifically deaminates tRNA^Arg2^. No eukaryotic tRNA substrates were found to be modified by *tadA*, except for yeast tRNA^Arg^ [16]. Because *tadA* is essential in *E. coli*, we used the *tadA* gene for further analysis.

**Table 1:** List of identified genes in the ASKA pooled-plasmid library enrichment.

Gene Name	Function	COG Classification ^a^	Essentiality ^b^
*tadA*	tRNA-Specific Adenine Deaminase	Translation	Essential
*galU*	Glucose-1-Phosphate Uridylyltransferase	Cell Envelope Biogenesis, Outer Membrane	Non-essential
*ydaC*	Protein Involved in Chromosome Maintenance/Rac Prophage Gene	DNA Replication, Recombination, and Repair	Non-essential
*fucU*	l-Fucose Mutarotase	Carbohydrate Transport and Metabolism	Non-essential
*mltF (yfhD)*	Membrane-Bound Lytic Murein Transglycosylase F	Cell Envelope Biogenesis, Outer Membrane	Non-essential
*ydeA*	Sugar Efflux Transporter	Carbohydrate Transport and Metabolism	Non-essential
*soxS*	DNA-Binding Transcriptional Regulator	Transcription	Non-essential
*yiiD*	Predicted Acetyltransferase	General Function Prediction Only	Non-essential
*nrdH (ygaN)*	Glutaredoxin-Like Protein	Post-Translational Modification, Protein Turnover, Chaperones	Non-essential ^c^

^a^ COG: Cluster of Orthologous Groups [23]; ^b^ These data were obtained from the Database of Essential Genes [15,24,25,26]; ^c^ The Database of Essential Genes classifies *nrdH* as an essential gene, but a knockout mutant is present in the KEIO collection [17].

The other eight genes that were isolated from the enrichment are listed as non-essential genes. However, among these genes, the essentiality of *nrdH* is ambiguous because, although Gerdes *et al.* classified *nrdH* as an essential gene [18], a knockout-mutant strain of *nrdH* has been constructed and is present in the KEIO non-essential gene-knockout collection [17]. Although *galU* is not essential for bacterial growth, this gene plays an important role in bacterial virulence, for example in *Francisella tularensis* [27]. *ydaC* (synonym: *rcbA*) encodes a protein that is involved in maintaining the integrity of the bacterial chromosome by lowering the steady-state level of double-strand breaks [28]. Overexpression of *ydaC* leads to increased resistance to erythromycin [10]. *mltF* encodes a membrane-bound lytic transglycosylase that is responsible for the release of 1,2-anhydromuropeptides from peptidoglycan [29]. MltF (YfhD) is located in the periplasmic space and is involved in organic solvent tolerance. However, the deletion or loss of *mltF* does not cause any loss of such tolerance [30]. SoxS is a DNA-binding transcriptional dual regulator. This regulator activates the transcription of the *soxRS* regulon, which is involved in the oxidative stress-defense system of *E. coli* [31]. Overexpression of *soxS* has also been reported to increase resistance to triclosan in *E. coli* [32]. This increased resistance is due to the expression control of genes involved in drug efflux, thereby leading to drug resistance [33]. Furthermore, modifications of lipopolysaccharide that are mediated by SoxS can protect *E. coli* against various compounds and increase resistance to multiple drugs [34]. The *yiiD* gene encodes a predicted acetyltransferase. Little information is available regarding this gene, but in a protein interaction study, Butland *et al.* revealed that *yiiD* interacts with an acyl carrier protein that is a key player in fatty-acid chain elongation [35].

### 2.2. Verification of tadA Overexpression-Induced Resistance to Xanthorrhizol in E. coli W3110 imp4213 and Bacillus subtilis 168, Site-Directed Mutagenesis, and Binding Study

Our results revealed that an essential gene, *tadA*, was a candidate that might be responsible for increasing the resistance of the *E. coli* W3110 *imp4213* ASKA pooled library to xanthorrhizol. The level of *tadA* gene overexpression was confirmed by cDNA analysis (Figure S1). *tadA* cDNA was increased approximately twofold compared to the control (Figure S1). We isolated plasmid pCA24N-*tadA* from the original ASKA library, re-introduced it into *E. coli* W3110 *imp4213*, and measured the MIC of xanthorrhizol against the resulting strain. *E. coli* W3110 *imp4213* (pCA24N-*tadA*) exhibited xanthorrhizol resistance, with an MIC of 128 µg/mL (Table 2). This MIC was not as high as that of the enriched library culture, a result that is likely due to the library being a mixed culture in which each clone contributes to the overall resistance towards xanthorrhizol.

**Table 2:** Xanthorrhizol minimum inhibitory concentration s of wild-type and mutant *tadA*-overexpressing strains.

Bacterial Strain	MIC (μg/mL)
*E. coli* W3110 *imp4213*	16
*E. coli* W3110 *imp4213* (pCA24N)	16
*E. coli* W3110 *imp4213* (pCA24N-*tadA*)	128
*E. coli* W3110 *imp4213* (pCA24N-*tadA* (A143V))	256
*E. coli* W3110 *imp4213* (pCA24N-*tadA* (F149G))	256
*Bacillus subtilis* 168	8
*B. subtilis* 168 (pHT255)	8
*B. subtilis* 168 (pHT255-*tadA*)	32

As described in the Introduction Section, xanthorrhizol is more effective against Gram positive bacteria, such as *B. subtilis*. To confirm whether *tadA* is involved in increasing resistance towards xanthorrhizol, we treated *B. subtilis* 168 overexpressing *tadA* with xanthorrhizol. We observed that xanthorrhizol MICs increased fourfold (Table 2). This result suggests that *tadA* may be one of the xanthorrhizol targets in the bacterial cell.

Site-directed mutagenesis was used to confirm that *tadA* was the xanthorrhizol target. Using docking analysis, we found that substitutions at Ala143 and Phe149 would be likely to have only a small effect on overall protein folding (Figure S2). Ala143 and Phe149 are located at the C-terminus of the *tadA* protein. The subsequent determination of xanthorrhizol MICs confirmed that the *tadA* mutants A143V and F149G confer twofold increased resistance towards xanthorrhizol (Table 2).

**Figure 2:**
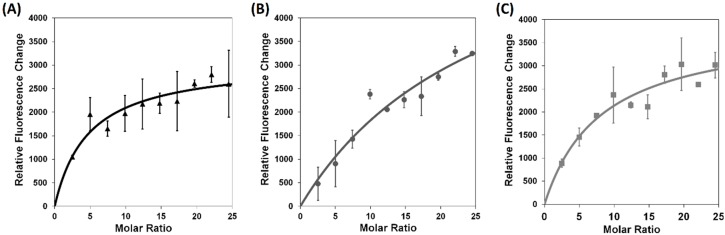
Interaction of Xanthorrhizol and *tadA* protein. Tryptophan quenching assay (**A**–**C**) were used to observe interaction between xanthorrhizol and *tadA* protein; (**A**) wild type *tadA*; (**B**) *tadA* (A143V); and (**C**) *tadA* (F149G). The assays showed a response, as revealed by fluorescence signal quenching, that xanthorrhizol bound to *tadA* protein. Tryptophan quenching assay demonstrated that binding capacity of xanthorrhizol to *tadA* mutants were lower than wild type, as shown by calculated Kd 19.04 μM, 109.7 μM, and 32.78 μM for wild type, A143V, and F149G, respectively.

The study of xanthorrhizol and *tadA* interaction was carried out using tryptophan quenching assay. Tryptophan quenching assay has been used to study binding activity of an enzyme inhibitor [36,37]. *tadA* protein contains three intrinsic tryptophan residues that make it suitable for quenching assay. The binding activity is indicated by the decreasing of fluorescence signal. In this study, 4 µM *tadA* protein was titrated with xanthorrhizol. Fluorescence titration demonstrated that xanthorrhizol was able to bind to the protein as revealed by fluorescence change with a Kd = 19.04 μM, 109.7 μM, and 32.78 μM for wild type *tadA*, A143V, and F149G, respectively (Figure 2). These results showed increasing Kd of xanthorrhizol in *tadA* mutants. However, the Kd for both *tadA* mutants was different even the MIC was the same. This could be because xanthorrhizol may have multiple intracellular targets that might contribute to its inhibition activity.

It should be noted that there have been no reports describing tRNA-specific adenosine deaminase as an antibiotic target. However, some information regarding antibiotics that target microbial RNA processing/editing enzymes is available. Among them, aminoacyl-tRNA synthetase (aaRS) is an essential enzyme and has for years been an attractive target for antibiotic development [38,39]. This enzyme catalyzes the synthesis of aminoacyl-tRNA, which plays an important role in protein biosynthesis. Targeting aaRS has also become one of the strategies to combat multidrug resistance Gram-negative bacteria [40]. Mupirocin, a natural product isolated from *Pseudomonas fluorescens*, is the only approved antibiotic targeting aaRS and is widely used as a topical antibiotic against *S. aureus* [41].

### 2.3. Modeling of the Binding Mechanism of Xanthorrhizol to Wild-Type and Mutant EcTadA

To identify the drug-resistance mechanism induced by the two mutations at sites 143 and 149, the interaction between *Ec*TadA and xanthorrhizol was explored in detail from energetic and structural perspective. The binding free energies of xanthorrhizol with WT *Ec*TadA and with the A143V and F149G mutants are −9.94 kcal/mol, −6.35 kcal/mol, and −6.69 kcal/mol, respectively (Table S1). Relative to wild-type *Ec*TadA, the contribution of ΔG_nonpolar_ is obviously decreased in the mutants (Table S1). The decrease in nonpolar interaction contribution can be caused by the loss of the van der Waals interaction contribution. The van der Waals contributions for both mutants decreased more than 5 kcal/mol relative to the wild type (Table S1).

**Figure 3:**
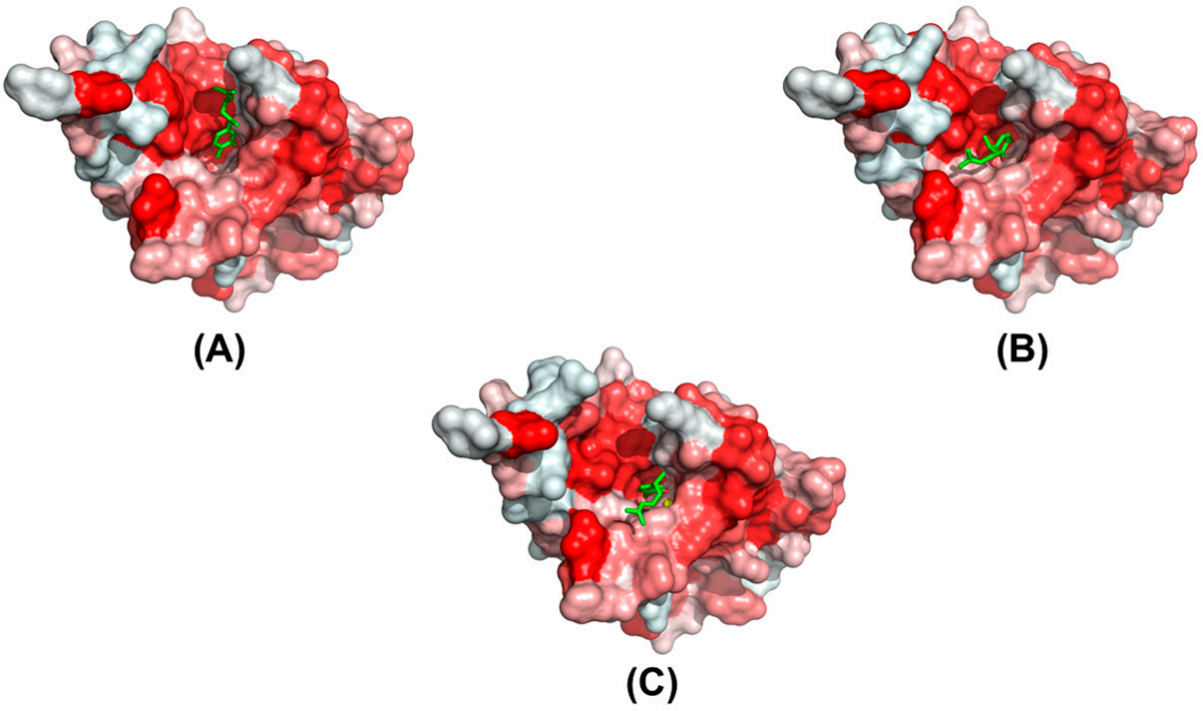
The hydrophobic surfaces of the wild-type and mutant *Ec*TadA complexes: (**A**) wild type; (**B**) A143V; and (**C**) F149G. Representative structures extracted from the MD trajectories were used. The proteins are shown in a surface representation, with the hydrophobic scale in red (high values are in dark red). The xanthorrhizol is shown with a green stick model.

**Figure 4:**
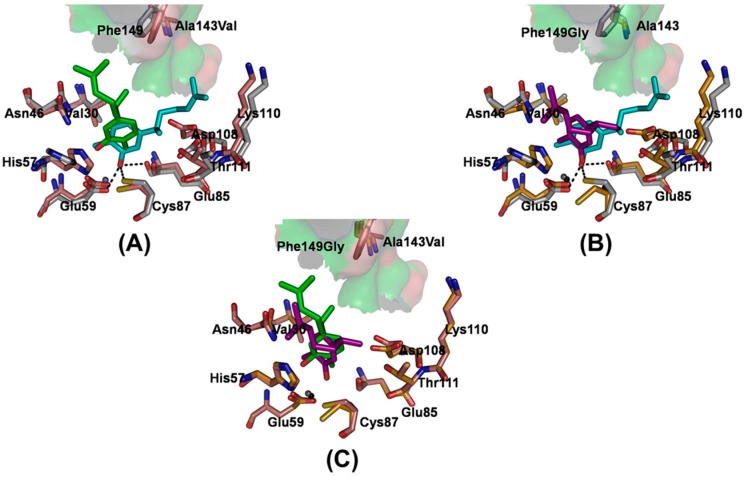
A superimposition of structures of the mutants relative to wild type complex. Representative structures of the mutants (**A**) A143V and (**B**) F149G aligned to the WT structure for a 20-ns snapshot taken from the complex trajectories. The carbon atoms of both mutants are colored salmon (A143V) and bright orange (F149G), and the carbon atoms of the WT system are colored light gray. The carbon atoms of xanthorrhizol are colored green (A143V) and purple (F149G). Xanthorrhizol and amino acid residues are displayed as sticks; (**C**) Representative structure of the A143V mutant aligned to the F149G structure. The xanthorrhizol (cyan) and the involved amino acid residues (light gray) are displayed as sticks in the structure of the xanthorrhizol and wild-type *Ec*TadA complex. The hydrogen bonds are displayed as black dashed lines.

Among the contributions of different energy components, the hydrophobic interaction, with a 50.82-kcal/mol contribution (Table S1), provides the main driving force for the binding of xanthorrhizol. A comparison of the hydrophobic surfaces (Figure 3) of the wild-type *vs.* either mutated *Ec*TadA reveals that both the hydrophobicity and the size of the hydrophobic pocket are influenced by the mutations. In the wild type, the phenolic group of xanthorrhizol was embedded well into the corresponding hydrophobic pocket containing the catalytic active site. In the A143V mutant, with the substitution from a small, hydrophobic alanine to a large, hydrophobic valine, the relative cavity size of the hydrophobic pocket is decreased. Furthermore, the phenolic group of xanthorrhizol is moved away from the favorable binding pocket (Figure 3B). In the F149G mutant, the substitution of Phe149 with a side chain-lacking glycine also results in an unfavorable hydrophobic interaction; as with the A143V mutation, the xanthorrhizol is forced to leave the favorable position (Figure 3C). A superimposition of the representative structures of the mutants relative to the wild-type complex showed the evidence that, in the mutants, the phenolic group of xanthorrhizol is forced to leave the original and favorable substrate-binding (Figure 4). Such a change in position can influence the hydrophobic/hydrophilic interactions between the phenolic group and the residues (Asp53, His57, Glu59, Glu85, Cys87, and Cys90) of the binding pocket. Furthermore, the hydroxyl group of xanthorrhizol has moved away from the cysteine at position 87 in the mutant complexes. Thus, the hydrogen bonding interaction between the phenolic hydroxyl group and the sulfur hydrogen of Cys87 is obviously affected.

Moreover, in Figure 4, in the two studied mutant versions of *Ec*TadA, xanthorrhizol is forced away from residues Asp108/Lys110/Thr111 (the favorable position) when compared with the wild-type system. In the mutant versions of *Ec*TadA, the increased nonpolar interactions of residues A143V and F149G with their surrounding residues are likely responsible for a decreased binding-pocket size, and the xanthorrhizol is thus moved away from the binding pocket. Furthermore, the dimethylethenyl group of xanthorrhizol is flipped away from the binding pocket in the two mutant systems compared to wild-type *Ec*TadA. As a result, the binding site of xanthorrhizol was moved by the mutations, and several important interactions were disturbed, including (1) the key hydrophobic interactions between the dimethylethenyl group and the surrounding hydrophobic residues; (2) the loss of the hydrogen bonds formed by the phenolic group with the side chains of Glu59/Glu85/Cys87; and (3) the gain of π-π stacking interactions between the phenolic benzene ring and His57, further decreasing binding affinity.

As described in the wet-experimental section, the mutants A143V and F149G each exhibit a twofold-decreased activity towards xanthorrhizol. Based on the above analysis, we speculate that the additional group in the side chain of each mutated residue will not form unfavorable contacts with the xanthorrhizol but will change its binding mode, which is in agreement with the root-mean-square deviation (RMSD) analysis of xanthorrhizol (see Supplemental Information Figure S3). As a result of this binding mode change, a π-π stacking interaction between the phenolic benzene moiety and His57 appears, and there is no obvious formation of the weaker hydrogen bonds between the hydroxyl group of Glu59, Glu85, and the sulfur hydrogens of Cys87.

## 3. Experimental Section

### 3.1. Chemicals, Bacterial Strains, and Plasmids

The ASKA clone (−) library and plasmid pCA24N were obtained from the National Bioresource Project, National Institute of Genetics, Japan. Plasmid pHT255 was obtained from MoBiTec GmbH (Goettingen, Germany). Bacterial strains used in this study are listed in Table 3. Xanthorrhizol was prepared in the Biomaterial Research Laboratory of Prof. Hwang at Yonsei University, Korea.

**Table 3:** Bacterial strains and primer pairs used for the site-directed mutagenesis of the *tadA* gene. The mutation site in the primer is underlined. Restriction enzyme site was written in bold letter.

Name	Description	Source
*E. coli* Strain		
DH5α	F^−^ endA1 glnV44 thi-1 recA1 relA1 gyrA96 deoR nupG Φ80d*lacZ*ΔM15 Δ(*lacZYA-argF*)U169, hsdR17(r_K_^−^ m_K_^+^), λ–	Laboratory Stock
W3110 *imp4213*	F^−^ λ^−^ rph-1 INV(rrnD, rrnE) Δ*imp::Tc^R^*	Laboratory Stock
W3110 *imp4213* (pCA24N)	W3110 Δ*imp* + pCA24N	Laboratory Stock
W3110 *imp4213* + pCA24N-*tadA*	*tadA* overexpression	This Study
W3110 *imp4213* + pCA24N-*tadA* (A143V)	*tadA* (A143V) overexpression	This Study
W3110 *imp4213* + pCA24N-*tadA* (F149G)	*tadA* (F149G) overexpression	This Study
*Bacillus* strain		
*B. subtilis* 168	*trpC2* (Trp^−^)	Laboratory Stock
*B. subtilis* 168 (pHT255)	*B. subtilis* 168 + pHT255	This Study
*B. subtilis* 168 (pHT255-*tadA*)	*tadA* overexpression	This Study
Primer		
A143V-F	GTGCGTGGCGTTGCTCAGTGAC	This Study
A143-R	TCATCCGCCAGTATTCCTTCC	This Study
F149G-F	AGTGACGGCTTTCGCATGCGCCGCCAG	This Study
F149-R	GAGCAACGCCGCGCACTCATCCGC	This Study
bstadA-F	CCGGC**TCTAGA**ATGACACAAGATG AACTTTATATGAAAGAAGC	This Study
bstadA-R	CCGGC**GACGTC**CTATTCAGACAAG TTTTTCCTGGC	This Study

### 3.2. ASKA Pooled-Plasmid Library Construction

The ASKA clone (-) library was obtained from frozen stocks, and individual clones were re-grown in 96-deep-well plates (Bioneer, Daejeon, Korea) containing LB medium supplemented with chloramphenicol (30 μg/mL) overnight at 37 °C in a Megagrowth incubator (Bioneer). All of the cultures were pooled, and plasmid DNA was extracted using the HighGene™ Plasmid Miniprep Kit (SolGent Co., Ltd., Daejeon, Korea). The pooled plasmids were introduced into *E. coli* W3110 *imp4213* via electroporation (Gene Pulser Xcell™ Electroporation System, BioRad Laboratories Inc., Hercules, CA, USA) and spread onto LB agar plates supplemented with chloramphenicol (30 μg/mL) and tetracycline (10 μg/mL). The plates were incubated at 37 °C overnight. The following day, *E. coli* W3110 *imp4213* cells harboring the ASKA pooled-plasmid library were scraped up and pooled into 10 mL of LB.

### 3.3. Xanthorrhizol-Resistance Library Enrichment and Screening

**Figure 5:**
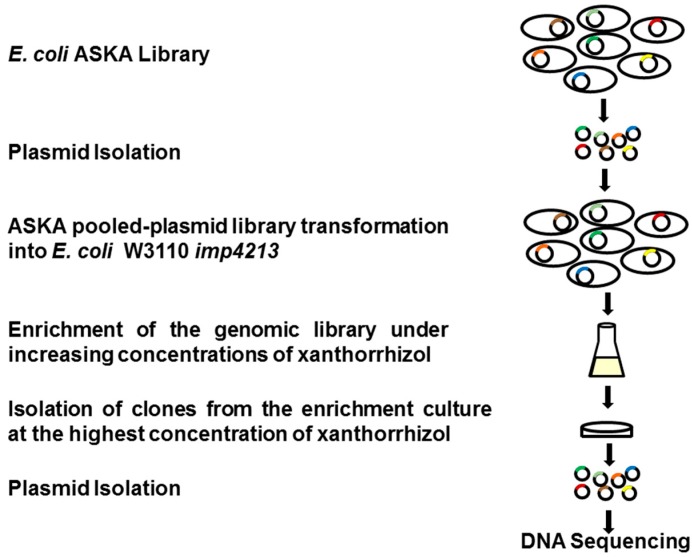
A scheme of the ASKA pooled-plasmid library enrichment screen for targets that confer xanthorrhizol resistance on *E. coli* W3110 *imp4213*. The ASKA plasmid library was isolated and re-introduced into competent *E. coli* W3110 *imp4213* cells by electroporation. The *E. coli* W3110 *imp4213* ASKA plasmid library clones were pooled and cultivated in LB medium containing chloramphenicol (30 µg/mL) in the absence or presence of xanthorrhizol (8–512 µg/mL). Cells cultured from the highest xanthorrhizol concentration were spread onto LB agar medium containing chloramphenicol (30 µg/mL), tetracycline (10 µg/mL) and appropriate concentrations of xanthorrhizol. The resulting colonies were randomly chosen for plasmid isolation followed by DNA sequencing analysis.

The library enrichment was done according to the procedure described by Yu *et al.* [11] with some modifications. The expression of the genes in the plasmid library was induced by adding IPTG at a final concentration of 100 µM. The first round of enrichment included xanthorrhizol concentrations of 0, 8, 16, 32, 64, and 128 μg/mL. The bacterial cultures were incubated in a TVS1 Bio-Photorecorder, a temperature-controlled shaking incubator (Advantec), at 37 °C for 20 h. The library culture that grew at the highest xanthorrhizol concentration was used as the inoculum for the second and third rounds of enrichment with increasing xanthorrhizol concentrations. These enrichments were repeated for a total of 3 independent experiments. After the third enrichment, the xanthorrhizol-enriched cell cultures were spread onto LB agar media supplemented with tetracycline (10 μg/mL), chloramphenicol (30 μg/mL), and an appropriate xanthorrhizol concentration. Individual colonies were chosen for plasmid isolation followed by DNA sequencing to identify the gene inserts. An overall scheme for the ASKA pooled-library screen is presented in Figure 5.

### 3.4. Verification of tadA Overexpression-Induced Resistance to Xanthorrhizol and Site-Directed Mutagenesis

To verify resistance towards xanthorrhizol, we isolated the pCA24N-*tadA* plasmid from the original ASKA clone and then re-introduced it into *E. coli* W3110 and *E. coli* W3110 *imp4213*. *E. coli* W3110 *imp4213* (pCA24N-*tadA*) was grown overnight in 3 mL of LB supplemented with tetracycline (10 μg/mL) and chloramphenicol (30 μg/mL). Approximately 1% of the culture was inoculated into fresh medium with the same antibiotics and grown in a shaking incubator at 37 °C to an OD_600nm_ of 0.5. Plasmid-insert expression was induced in the culture by the addition of IPTG at a final concentration of 100 μM. The cells were allowed to grow for 6 h. This culture was then used as the inoculum for an MIC assay. The microdilution broth method [42] was employed to determine the MIC of xanthorrhizol against *E. coli* W3110 *imp4213* (pCA24N-*tadA*). The inoculum was adjusted to an OD_625nm_ of 0.08, and the clone was tested against a range of xanthorrhizol concentrations (0, 4, 8 16, 32, 64, 128, 256, and 512 μg/mL). The MIC determination was performed in duplicate and repeated for a total of 3 independent experiments.

We predicted that substitutions of the amino acids at position A143 to V143 and at position F149 to G149 would increase resistance towards xanthorrhizol. Thus, we designed primer pairs to introduce these substitutions (Table 3). Plasmid pCA24N-*tadA* from the ASKA clone was used as the template. Site-directed mutagenesis was performed using the Exchange™ Site-Directed Mutagenesis Kit (Enzynomics, Daejeon, Korea). The mutant plasmid was introduced into chemically competent DH5α (Enzynomics) and spread onto LB supplemented with chloramphenicol (30 µg/mL). The culture was then incubated overnight at 37 °C. Colonies from each transformation were chosen for plasmid isolation followed by DNA sequencing analysis to confirm the mutation. DNA sequencing analysis was performed by SolGent Co. Ltd. Plasmid isolation was performed using the HiGene™ Plasmid Isolation Kit (SolGent Co. Ltd.). All mutants were tested for their resistance to xanthorrhizol by measuring MICs. To measure the MICs of the *tadA* mutants, we used the same protocol as described above. This experiment was performed in duplicate and repeated for a total of 3 independent experiments.

### 3.5. tadA Overexpression in B. subtilis 168

*B. subtilis tadA* gene was amplified from genomic DNA as a template employing PCR amplification. Primer used in the amplification was provided in Table 3. The amplified gene was digested and inserted into pHT255 vector plasmid at *Xba*I and *Aat*II restriction site. Construction pHT255 carrying *tadA* gene was done in *E. coli* DH5α. Subsequently, pHT255 and pHT255-*tadA* was introduced into naturally competent *B. subtilis* 168 using Groningen method [43].

*B. subtilis* 168 harboring empty plasmid (pHT255) and *tadA* gene (pHT255-*tadA*) was grown on LB agar plate supplemented by Cm (5 µg/mL) overnight at 37 °C. On the following day, about 1-loop colony of the cultures were inoculated into a LB + Cm (5 µg/mL) and grown until OD_600nm_ of 0.7–0.8. Subsequently, 1 mM IPTG was added to induce *tadA* gene expression. The cultures continued to grow for another 6 h. These induced cultures were used as inoculum for MIC determination. MIC determination was done as mentioned above. This experiment was performed in duplicate and repeated for a total of 3 independent experiments.

### 3.6. Xanthorrhizol-tadA Binding Study

*E. coli* strains producing wild-type *tadA* or the *tadA* (A143V) and *tadA* (F149G) mutants were grown overnight at 37 °C in LB supplemented with chloramphenicol (30 µg/mL). About 1% of each culture was inoculated into fresh medium and incubated for approximately 2–3 h until the OD_600nm_ reached 0.5. The cultures were then induced by the addition of 100 µM IPTG and allowed to grow for an additional 8 h. To purify his-tagged *tadA* protein, cells were harvested and lysed by sonication. Proteins were bound to agarose resin (His•Bind Agarose Resin (Ni-IDA), Elpis Biotech, Daejeon, Korea) and purified through column chromatography as described in the kit protocol (His•Bind Agarose Resin (Ni-IDA) Elpis Biotech). Protein was desalted by using PD-10 desalting columns following the protocols described by the manufacturer (GE Healthcare Bio-Sciences AB, Uppsala, Sweden).

A tryptophan quenching assay was used to study the xanthorrhizol-*tadA* interaction. *tadA* (4 µM) in 50 mM Tris-Cl (pH 8) and 10% glycerol was titrated with 2 µL xanthorrhizol (5 mM) and was then immediately excited at 295 nm; the emission was recorded from 300 to 500 nm using a FluoroMate FS-2 fluorescence spectrophotometer (Scinco Co. Ltd., Seoul, Korea). The excitation and emission monochromator slit widths were 2.5 nm. The fluorescence decrease (*F*_0_ − *F*) caused by the addition of xanthorrhizol was fitted to the equation (*F*_0_ − *F*) = Δ*F*_max_ × *[I]*/(*K_d_* + *[I]*) to construct the binding curve. The assay was repeated at least three times.

### 3.7. Computational Methods

#### 3.7.1. Molecular Docking

The *Ec*TadA crystal structure (PDB ID: 1Z3A) was used as the input for xanthorrhizol docking using two different docking programs, GLIDE [44] and GOLD [45]. The substrate and cofactor (tRNA and Zn^2+^, respectively) were imported from the crystal structure by structural superposition [46]. Proton addition was performed with the corresponding algorithms implemented in the different docking programs. In GLIDE (version 5.8), the considered search volume was confined within a cube of 20 Å^3^. A total of 5000 poses per xanthorrhizol were retained for the initial phase of docking, and the best 400 poses per xanthorrhizol were retained for energy minimization. In GOLD (version 5.1), the search was confined within a 10-Å-radius sphere around the center defined above with the slowest and most accurate genetic algorithm search options, generating 10 poses per xanthorrhizol. All 4 built-in scoring functions in GOLD (ChemPLP, GoldScore, ChemScore, and ASP) were used in parallel docking simulations. Finally, the xanthorrhizol structure was built using the Maestro software [47] included in the Schrödinger Suite 2011 package and was energy-minimized using Macromodel [48] with the OPLS_2005 force field.

#### 3.7.2. Molecular Dynamics Simulations

Molecular dynamics simulations were performed using the program Q [49] with the OPLS-AA force field [50]. Subsequently, the binding free energy was calculated as described in the supplementary materials. The parameters needed for xanthorrhizol that were not present in the original version of the force field were retrieved from the automatic parametrization performed with Macromodel [48]. Spherical boundary conditions were used with a 20-Å-radius simulation sphere centered on the same point as defined for the docking calculations. This sphere was solvated with TIP3P [51] water molecules and subjected to polarization and radial constraints according to the surface-constrained all-atom solvent (SCAAS) model [49,52] at the sphere surface to mimic the properties of bulk water. Non-bonded interactions were calculated explicitly up to a 10-Å cutoff, except for the xanthorrhizol atoms, for which no cutoff was used. Beyond the cutoff, long-range electrostatics were treated with the local reaction field multipole-expansion method [53]. All solvent bonds and angles were constrained with the SHAKE algorithm [54], and a 1-fs molecular dynamics (MD) step size was used. The MD simulations were performed at a temperature of 298 K. Non-bonded pair lists were updated every 25 steps, and the same interval was used to sample the xanthorrhizol-surrounding residue interaction energies.

## 4. Conclusions

By enriching an *E. coli* W3110 *imp4213* ASKA pooled-plasmid library, we found that TadA may be involved in the mechanism of xanthorrhizol action. We have shown that xanthorrhizol targets TadA and, more importantly, that tRNA-specific adenosine deaminase may be a new target for antibiotic development in the future.

## Supplementary Materials

Supplementary materials can be accessed at: http://www.mdpi.com/1420-3049/20/09/16290/s1.
