# Supplementary material for: Escherichia coli ASKA Clone Library Harboring tRNA-Specific Adenosine Deaminase (tadA) Reveals Resistance towards Xanthorrhizol
Source: Molecules. 2015 Sep 9;20(9):16290–305. doi: 10.3390/molecules200916290 (PMC6331797; doi:10.3390/molecules200916290)
Supplement: Supplementary file 1 [file molecules-20-16290-s001.pdf]

Supplementary Information

1. Expression Confirmation by *tadA* cDNA Analysis

To confirm the expression of *tadA* gene in high concentration of xanthorrhizol, total RNA extraction was performed using RNAiso Plus (Takara Bio Inc., Shiga, Japan). Approximately 2 mL of culture was centrifuged at 10,000× *g* for 2 min. The pelleted cells were homogenized in 1 mL of RNAiso Plus solution and incubated at room temperature for 5 min, followed by the addition of 200 µL of chloroform. The solution was vortexed and centrifuged at 12,000× *g* at 4 °C for 15 min. Approximately 400 µL of the aqueous phase was transferred into a new tube. To precipitate the RNA, 1 volume of isopropanol was added, and the mixture was incubated at room temperature for 30 min. RNA was collected by centrifugation at 12,000× *g* at 4 °C for 15 min. The supernatants were discarded, and the pellets were washed with 70% ethanol in DEPC-treated water and air-dried to remove excess ethanol. The RNA was eluted with DEPC-treated water.

cDNA synthesis was performed with TOPscript™ One-step RT PCR DryMix (Enzynomics, Daejeon, Korea). The PCR mixture was prepared as follows: 1 tube of TOPscript™ One-step RT PCR DryMix, 2 µL of the RNA template (100 ng/µL), 1 µL each of the *tadA* forward and reverse primers (10 pmol/µL), and DEPC water up to a final volume of 20 µL. The mixture was incubated at 55 °C for 30 min. The PCR cycle was run as follows: an initial denaturation for 10 min at 95 °C; 35 cycles of a 30-s denaturation at 95 °C, a 30-s annealing at 55 °C, and a 30-s elongation at 72 °C; and a final 5-min elongation at 72 °C. The reaction was subsequently stored at 4 °C. The resulting PCR products were electrophoretically separated on a 1% (*w*/*v*) agarose gel. cDNA band quantity was measured by GelQuant.NET software version 1.8.2. We used GelQuant.NET software provided by biochemlabsolutions.com. cDNA band quantity from treated library was compared to control by one-way analysis of variance followed by Dunnett's test (SigmaPlot 12, Systat Software Inc., San Jose, CA, USA); * $p < 0.05$ was considered significant.

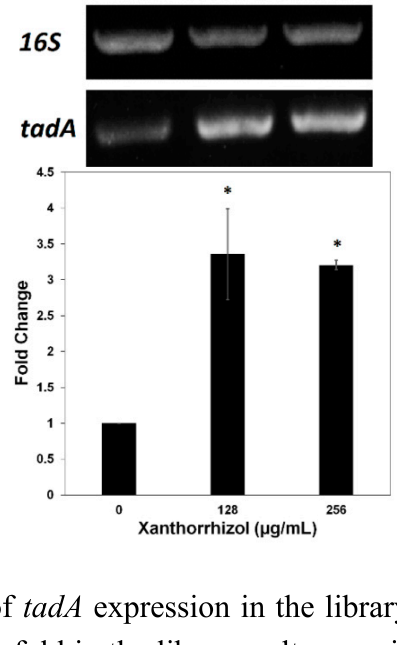

Figure S1. cDNA analysis of *tadA* expression in the library culture. The concentration of *tadA* increased more than twofold in the library culture enriched with a high concentration of xanthorrhizol. 16S rDNA was used as a control. cDNA fold change from treated library was compared to control by one-way analysis of variance followed by Dunnett's test; * $p < 0.05$ was considered significant.

2. Computational Methods

Binding Affinity Calculations

The convergence was estimated by dividing the whole trajectory into two halves of equal length, and the associated errors were estimated as the difference between the average energies of the first half and second half of the data-collection phase. Each xanthorrhizol:*Ec*TadA docking pose (for both wild-type TadA and its mutants) was subjected to five independent MD simulations using the same conditions but with different initial random velocities. *Ec*TadA atoms outside the simulation sphere were restrained to their initial positions and only interacted with the system through bonds, angles, and torsions. The equilibration phase lasted for 0.5 ns, during which all solute heavy atoms were restrained and gradually released as the temperature increased. No restraints were applied during the final 140 ps, preceding the subsequent data-collection phase that lasted for 2.5 to 5 ns, depending on the convergence achieved. It may be noted here that the average convergence error for the estimated binding free energies was only ±0.53 kcal/mol. Likewise, each xanthorrhizol in wild-type *tadA* and its mutants was subjected to five independent MD simulations in water following the same scheme but with different initial random velocities. After a similar equilibration scheme, this calculation was followed by a 1-ns unrestrained MD production phase, where a weak harmonic restraint (10 kcal/mol·Å^2) was applied to the center of mass of the xanthorrhizol. Subsequently, the binding free energies were calculated using the linear interaction energy (LIE) method [1,2]. Here, the binding affinities (ΔG_{bind}) were estimated on the basis of the difference (Δ) in the average xanthorrhizol-surrounding interaction energies (U) extracted from the MD simulations of the xanthorrhizol in the two states referred to above, *i.e.*, in water and embedded in the active site of the solvated *tadA*. The nonpolar (U_{vdW}) and polar (U_{el}) interaction energies are treated separately, and their difference is scaled by different scaling factors, α and β:

$$\Delta G_{bind} = \alpha\Delta\langle U_{vdW}\rangle + \beta\Delta\langle U_{el}\rangle + \gamma \quad (1)$$

The constant α has been empirically set to 0.18, while β varies depending on the properties of xanthorrhizol [2,3]. Unlike α and β, the parameter γ generally depends on the protein-binding site (its hydrophobicity or desolvation cost) [4], and it is treated as a free parameter and empirically adjusted. An electrostatic correction term, ΔG_{corr}, was calculated for the interactions of charged xanthorrhizol with distant, neglected ionized groups that were neutralized in the simulations as follows:

$$\Delta G_{corr} = \sum_{p}\sum_{l}\frac{q_p q_l}{\varepsilon r_{pl}} \quad (2)$$

where q_p is the formal charge of the neglected ionic group, while q_l is the partial charge of the xanthorrhizol atom; the effective dielectric constant ε was set to 80 [5]. The offset parameter γ was optimized to minimize the absolute error between the calculated and experimental binding free energies, and this optimization was initially only based on xanthorrhizol with a known crystallographic binding mode (it does not affect relative binding free energies).

3. Computational Results

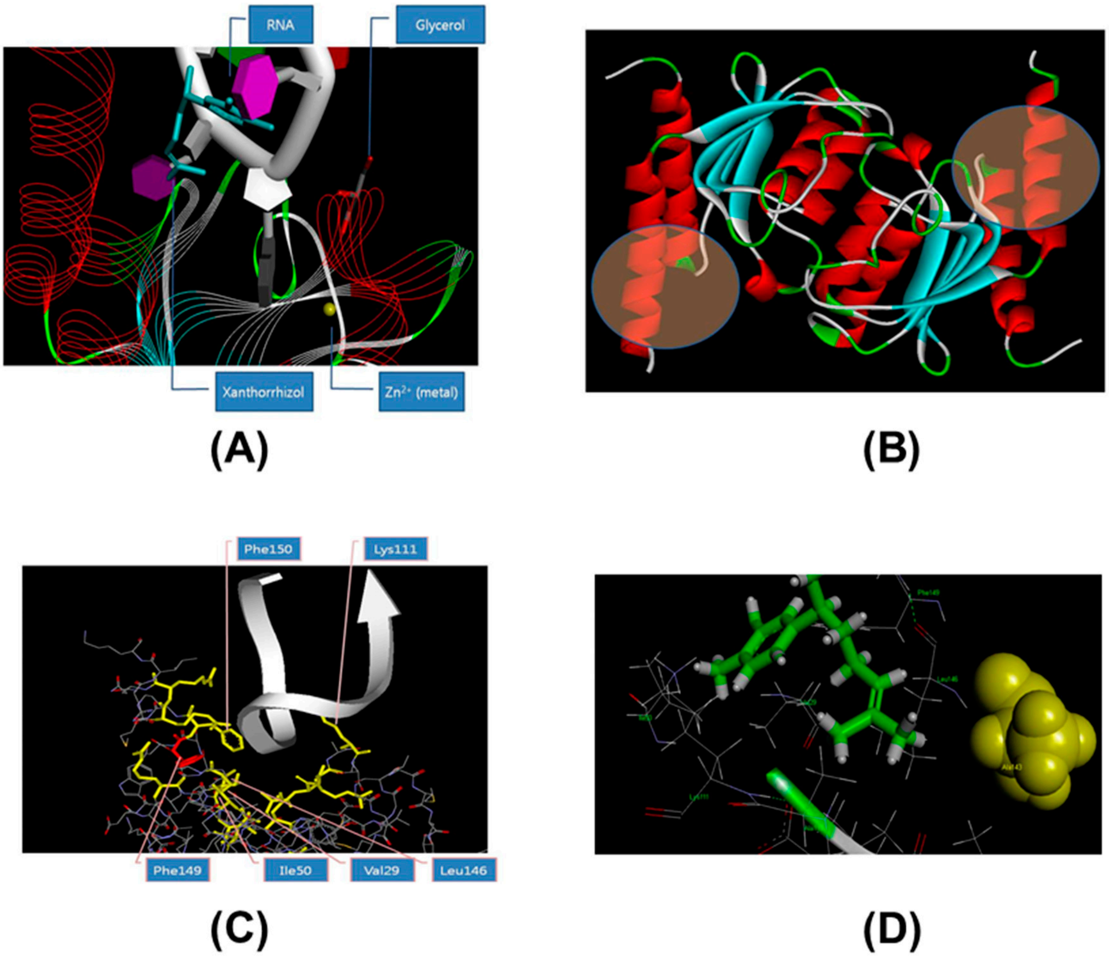

Figure S2. Docking analysis of TadA-xanthorrhizol interactions and site-directed mutagenesis predictions. (**A**) Comparison and superimposition of the tRNA-binding site and xanthorrhizol. The xanthorrhizol-binding site is apart from the Zn^{2+}-binding site. Xanthorrhizol binding prevents the reaction from progressing; (**B**) Dimer formation by the TadA protein. The regions marked by circles are the ligand-binding site and the C-terminus. If a mutation is introduced at this site, it will exert a small effect on overall protein folding; (**C**) Phe149 is marked in red, and tRNA is shown as an arrow-shaped ribbon. Phe149 is involved with xanthorrhizol binding but not with tRNA binding; (**D**) Ala143 (yellow) provides a large entrance to the active site. The mutation of Ala143 reduces the size of the entrance cavity.

Table S1. The calculated binding free energy and its components (kcal/mol) of the wild-type (WT) and two mutant (A143V and F149G) *Ec*TadA versions with xanthorrhizol. All energies are in kcal/mol. The computed binding free energy ($\Delta G_{bind} = G_{complex} - G_{EcTadA} - G_{xanthorrhizol}$) is composed of the following energy values: the hydrophobic interaction ($\Delta G_{nonpolar} = \Delta E_{vdw} + \Delta G_{sol\text{-}np}$), the polar interaction ($\Delta G_{polar} = \Delta E_{Eelec} + \Delta G_{sol\text{-}elec}$), the intermolecular electrostatic interactions (ΔE_{Eelec}), the large desolvation penalties ($\Delta G_{sol\text{-}elec}$), the total polar interaction ($\Delta G_{polar} = \Delta E_{Eelec} + \Delta G_{sol\text{-}elec}$), and the van der Waals contribution (ΔE_{vdw}).

	WT	A143V	F149G
ΔE_{elec}	−14.49	−2.24	−11.70
ΔE_{vdW}	−44.87	−39.28	−37.46
ΔE_{MM}	−59.36	−41.51	−49.16
$\Delta G_{sol\text{-}np}$	−5.95	−5.77	−5.61
$\Delta G_{sol\text{-}elec}$	30.49	16.84	25.29
ΔG_{sol}	24.54	11.07	19.67
ΔG_{polar}	16.01	14.61	13.59
$\Delta G_{nonpolar}$	−50.82	−45.05	−43.07
ΔH_{bind}	−34.82	−30.44	−29.48
$T\Delta S$	−24.88	−24.09	−22.80
ΔG_{bind}	−9.94	−6.35	−6.69

RMSD Analysis

To determine whether the studied system is at equilibrium, the RMSDs of the Cα atoms of the active-site residues (residues within 5 Å of xanthorrhizol), all the backbone atoms of the protein and the heavy atoms of xanthorrhizol were monitored relative to the starting structure and plotted in Figure S3. As illustrated in Figure S3A,B, the RMSDs of the binding pocket and the protein showed generally small fluctuations of 10 ns, indicating that the studied system reached equilibrium after 10 ns. Based on the time evolution of the RMSD of the active site, the A143V strain has the largest structural fluctuation of all of the studied systems. The averaged RMSD values of the active-site Cα atoms from 10 to 30 ns in the MD simulations were 0.65 Å, 1.31 Å and 0.94 Å for wild type, A143V, and F149G, respectively. Additionally, monitoring the RMSD changes of the heavy atoms of xanthorrhizol shown in Figure S3A,B revealed that the RMSD of xanthorrhizol for the wild-type system had a very small value that remained stable throughout all MD simulations. However, the two mutant systems had relatively higher RMSD values for xanthorrhizol and showed larger fluctuations, especially for the F149G mutant complex, indicating that the two mutations influence the binding mode of xanthorrhizol and render its binding less stable. To further explore the conformational changes of the protein induced by the two mutations, a root mean square fluctuation (RMSF) analysis was performed. Figure S3C shows that the flexibility of four regions (the residues from 53 to 62, 80 to 93, 102 to 113, and 140 to 152) in the mutants was obviously increased compared to the same regions of wild-type *Ec*TadA. As described previously [6–8], key residues in *Ec*TadA, including Asp53, HiS37, Glu59, Cys87, and Cys90, can mediate important interactions with xanthorrhizol and tRNA(A_{34}); all of these residues are within the four regions described above, suggesting that mutations in these regions will make *Ec*TadA more flexible and weaken its interaction with xanthorrhizol.

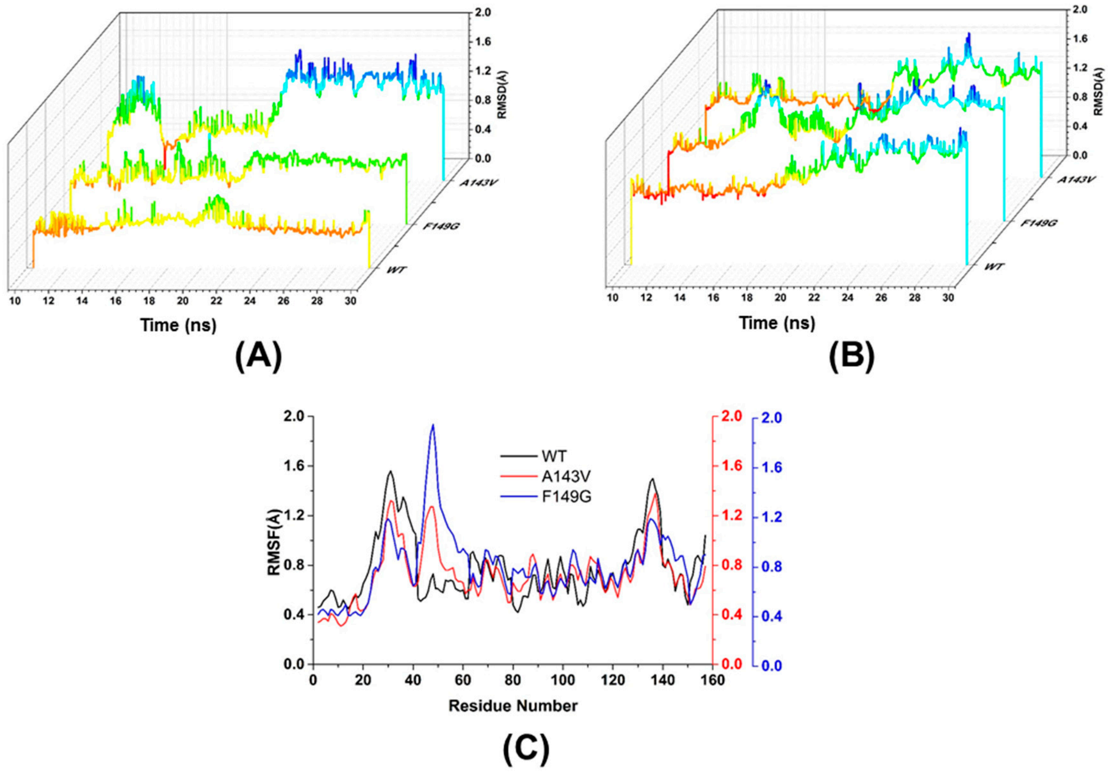

Figure S3. Equilibrium monitoring of the molecular dynamics (MD) trajectories: (**A**) time evolution of the root-mean-square deviation (RMSD) of the Cα atoms for the residues within approximately 5 Å of xanthorrhizol; (**B**) time series of the RMSD of the protein backbone atoms; and (**C**) calculated root-mean-square fluctuations (RMSFs) of the Cα atoms relative to the initial structure.

References

1. Åqvist, J.; Medina, C.; Samuelsson, J.-E. A new method for predicting binding affinity in computer-aided drug design. *Protein Eng.* **1994**, *7*, 385–391.
2. Hansson, T.; Marelius, J.; Åqvist, J. Ligand binding affinity prediction by linear interaction energy methods. *J. Comput. Aided Mol. Des.* **1998**, *12*, 27–35.
3. Almlöf, M.; Carlsson, J.; Åqvist, J. Improving the Accuracy of the Linear Interaction Energy Method for Solvation Free Energies. *J. Chem. Theory Comput.* **2007**, *3*, 2162–2175.
4. Almlöf, M.; Brandsdal, B.O.; Åqvist, J. Binding affinity prediction with different force fields: Examination of the linear interaction energy method. *J. Comput. Chem.* **2004**, *25*, 1242–1254.
5. Marelius, J.; Hansson, T.; Åqvist, J. Calculation of ligand binding free energies from molecular dynamics simulations. *Int. J. Quantum Chem.* **1998**, *69*, 77–88.
6. Kim, J.; Malashkevich, V.; Roday, S.; Lisbin, M.; Schramm, V.L.; Almo, S.C. Structural and Kinetic Characterization of Escherichia coli TadA, the Wobble-Specific tRNA Deaminase. *Biochemistry* **2006**, *45*, 6407–6416.
7. Luo, M.; Schramm, V.L. Transition State Structure of *E. coli* tRNA-Specific adenosine deaminase. *J. Am. Chem. Soc.* **2008**, *130*, 2649–2655.
8. Wolf, J.; Gerber, A.P.; Keller, W. *tadA*, an essential tRNA-specific adenosine deaminase from *Escherichia coli*. *EMBO J.* **2002**, *21*, 3841–3851.
